# Incidental Finding in Pre-Orthodontic Treatment Radiographs of an Aural Foreign Body: A Case Report

**DOI:** 10.3390/children9030421

**Published:** 2022-03-15

**Authors:** Cinzia Maspero, Andrea Abate, Francesco Inchingolo, Claudia Dolci, Maria Grazia Cagetti, Gianluca Martino Tartaglia

**Affiliations:** 1 Department of Biomedical, Surgical and Dental Sciences, School of Dentistry, University of Milan, 20100 Milan, Italy; andreabate93@gmail.com (A.A.); maria.cagetti@unimi.it (M.G.C.); gianluca.tartaglia@unimi.it (G.M.T.); 2 Fondazione IRCCS Cà Granda, Ospedale Maggiore Policlinico, 20100 Milan, Italy; 3 Department of Interdisciplinary Medicine, Università degli Studi di Bari “Aldo Moro”, 70124 Bari, Italy; f.inchingolo@icloud.com; 4 Department of Biomedical Sciences for Health, Università degli Studi di Milano, 20133 Milan, Italy; claudia.dolci@unimi.it

**Keywords:** aural, foreign body, orthodontics, lateral cephalogram, orthopantomography

## Abstract

The presence of foreign bodies in the external auditory canal of young patients may cause, if left untreated, severe permanent damage to the adjacent anatomical structures, and infections. A 10-year-old patient with an intellectual disability underwent orthodontic evaluation. An aural radiopaque finding was visible in the lateral cephalogram and in the orthopantomography. The patient’s mother reported that her son never showed any ear discomfort, except for a mild hearing impairment that was never investigated. The patient was referred to an ear, nose and throat (ENT) specialist that removed the foreign body located in the left external auditory meatus. The careful evaluation of dental radiographs, including pre-orthodontic and interim orthodontic radiographs, may help to identify silent incidental findings that may otherwise lead to severe complications if left untreated.

## 1. Introduction

Foreign bodies (FBs) inside the external auditory canal and in the nasal cavity are a common finding in pediatric patients. These objects are not meant to be there and could cause harm without medical attention [1]. The EAC is an oval tube from the outer to the middle ear. Due to its sigmoid shape, foreign bodies can readily become lodged, particularly at the bony isthmus where the canal narrows at the bony cartilaginous junction, increasing the difficulty of removal [2].

Sometimes a child may put an object into another child’s ear during play. Children with intellectual impairments such as attention deficit hyperactivity disorder (ADHD) present an increased risk of self-placement of foreign bodies into their ears [3,4]. Self-inserted foreign bodies needing the intervention of an ear, nose and throat specialist is a relatively common event [4]. Most common findings include jewelry products, followed by cotton swabs, paper products, seeds, small batteries, buttons, toys or insects [5]. In many cases, patients with foreign bodies in the ear are asymptomatic, and in children the foreign body is often incidental [6,7,8]. Other patients may present with pain, symptoms of otitis media, hearing loss, or a sense of ear fullness [1].

A 5-year retrospective study reviewed cases of foreign bodies inserted in the nose, throat and the ear. The study underlined that 59.9% of 594 cases analyzed comprised foreign bodies in the ear [9]. 

In several large case series focusing on children, researchers found that 75 percent of patients with ear foreign bodies were younger than eight years old [7,8,10,11]. In one series, 30 percent of patients required general anesthesia to facilitate the removal of an ear foreign body; the majority of those patients were younger than seven years [4]. 

Panoramic radiograph is the preferred diagnostic tool for the first evaluation of maxillofacial structures in dentistry. Its use is preferred to other exams because of its relatively low radiation dosage and its capability of detecting a broad spectrum of maxillofacial alterations, such as large lesions, cysts and tumors [12]. The American Dental Association Council on Scientific Affairs and the U.S. Food and Drug Administration recommend the first radiographic examination in pediatric patients to be taken after the first permanent tooth erupts (around 6–7 years) to assess growth and development [13]. This age corresponds to the period of greatest discovery of foreign bodies in the external auditory canal. 

More than 90% of orthodontists routinely request radiographs for their treatment planning, including panoramic and lateral radiographs [14].

Orthodontists frequently request lateral cephalograms for cephalometric reasons, and in these cases incidental findings could sometimes go unnoticed [14].

The frequency with which a clinician may detect incidental findings such as pathology and abnormalities in a patient needing orthodontic treatment is of particular interest to orthodontists, because in many cases they may require medical or odontological management [15]. Bondermark et al. [15] reported that 8.7% of orthodontic patients exhibited the incidental finding of pathology or abnormality; the most common findings included idiopathic osteosclerosis, alterations regarding the mucous membrane of the maxillary sinuses, periapical lesions, marginal bone loss, and odontomas that often require dental management. The authors [14,15] reported a low percentage (0.16%) of cases of incidental findings of a foreign body, but they emphasized the importance for the orthodontist to carefully analyze the pre-orthodontic treatment radiographs not only from an orthodontic point of view. In specific cases such as craniofacial malformations, impacted teeth, etc., a three-dimensional imaging technique such as cone beam computed tomography (CBCT) is needed [16,17,18]. Some research studies have shown that incidental findings observed in CBCT scans can report a frequency of up to 92% [19,20]. Although the use of CBCT imaging has been increasing during the last year for diagnosis and treatment planning purposes in orthodontics, it is not yet a routine diagnostic method because of the amount of radiation exposure.

Nevertheless, only two cases of incidental findings of foreign bodies discovered through panoramic radiographs during dental examinations are described in the literature at the moment [21,22]. Only one of them was inside the external auditory canal. The other object was an impacted earring clip inside the earlobe [21]. The limited number of foreign bodies reported as incidental findings in dental radiographs may allegedly be due to a sub-diagnosis of this condition. Svider et al. correlated the most frequently foreign objects inserted into the EAC with age and sex [23]. The most frequently found foreign objects in pediatric patients are fashion jewelry pieces, crayons, pieces of chalk, batteries and pencils [23]. All of them are radiopaque and should be clearly visible in routine radiographs.

In the patient described in this paper, the long-standing foreign body was detected as an incidental finding in a pre-treatment lateral cephalogram prescribed for orthodontic evaluation and then confirmed by the same finding in the patient’s panoramic radiograph.

## 2. Case Report

A 10-year-old male patient with a mild intellectual disability was referred to the Department of Biomedical Surgical and Dental Sciences of Fondazione IRCCS Cà Granda, Ospedale Maggiore Policlinico, Milano, for orthodontic evaluation.

The patient’s mother reported no significative recent general health changes. 

**Figure 1:**
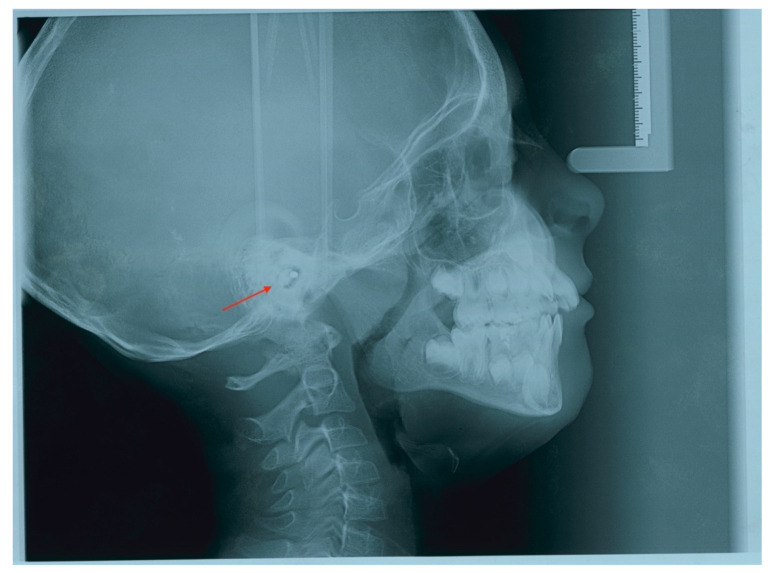
Lateral cephalogram showing a foreign body in the auditory canal.

Chief complaints of the patient’s mother included crowding and misalignment in both jaws, mouth breathing and snoring. After a carefully clinical evaluation performed by the orthodontist, the presence of maxillary hypoplasia which resulted in a posterior crossbite and a serious lack of space for the eruption of permanent later incisors and canines was reported. A concomitant mandibular shift on the right due to the maxillary transverse deficiencies was present. The maximum mouth opening (MMO) was 40.37 and considered normal. For the aforementioned reasons, the need of a prompt orthopedic/orthodontic treatment was considered necessary to re-establish a normal craniofacial growth pattern. Due to the patient’s general health conditions, his dentist referred him for orthodontic evaluation in a hospital that provides orthodontic treatment for patients with special needs. When the patient first came to the special care orthodontic department with the radiographs prescribed by his general dentist, the lateral cephalogram revealed an ovoid radiopaque shape at the level of the external auditory meatus. (Figure 1) 

**Figure 2:**
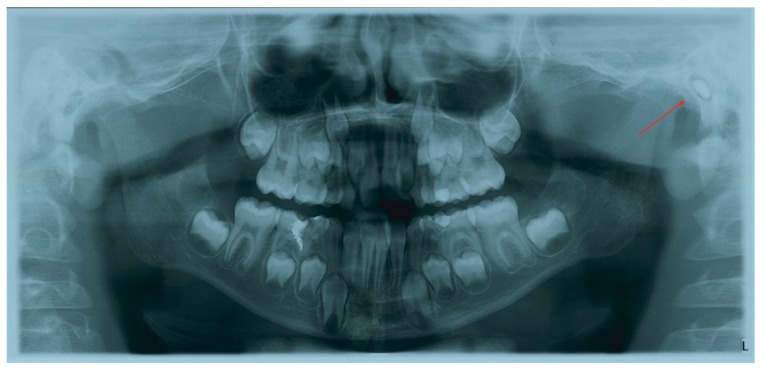
Orthopantomography showing foreign body in the left external auditory canal.

A similar radiopaque object was also noted on the contextually taken panoramic radiograph (Figure 2), apparently inside the left external auditory canal (EAC).

**Figure 3:**
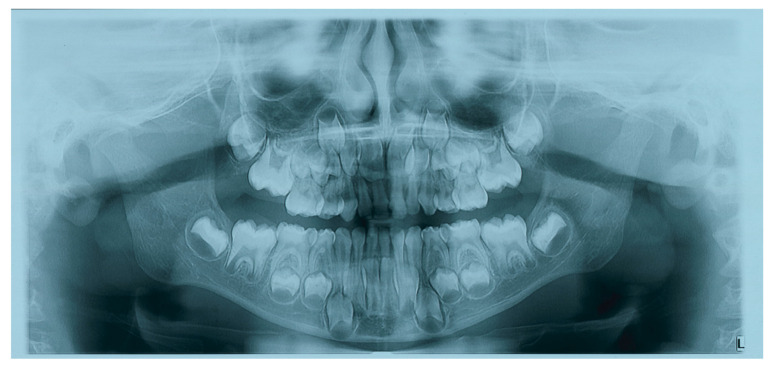
Previous orthopantomography showing no object in the left auditory canal.

The contour of the radiopaque finding appeared well defined with apparently no communication to the normal bony anatomical structures close to it with similar radiopacity. The previous radiographic records of the patient (a panoramic radiograph taken about two years before) showed no sign of any radiopacity sign inside the EAC (Figure 3). This finding was discussed with the patient’s mother, she reported that her son never showed discomfort or signs of infection to the ear except for a hearing impairment that the child developed about two years ago but was never investigated by his physician nor by other doctors that visited him.

Even though the patient did not recall having inserted anything in his ear, either voluntarily or accidentally, the Orthodontist decided to refer the young patient to a specialist of the Pediatric Otolaryngology Department of the same hospital.

**Figure 4:**
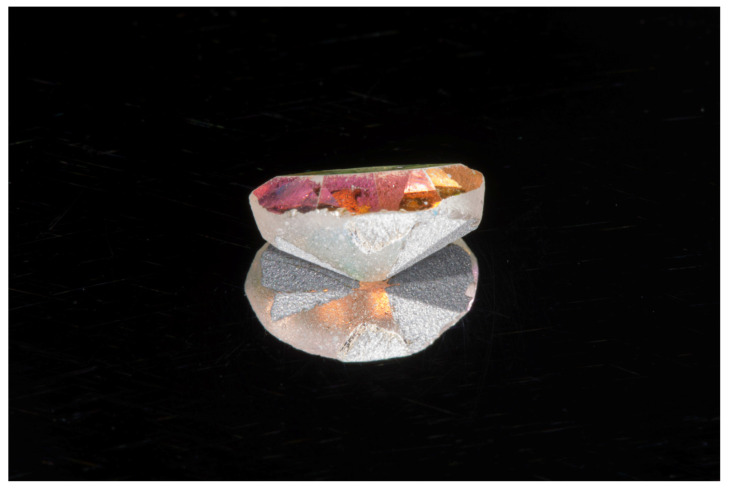
The foreign body removed from the external auditory canal.

An object was embedded in the cerumen. The ENT specialist grasped and removed the object from the auditory canal using an alligator forceps under mild sedation because of the intellectual disability of the patient. Cerumen and debris were removed with an otologic suction and loop curette. The tympanic membrane was intact after removal, and there was no significant trauma to the external canal. The foreign object was determined to be a piece of fashion jewelry with a metallic base (0.65 mm length, 0.4 mm width, 0.25 mm thickness) (Figure 4). The patient did not remember placing anything into his ear accidentally. After the removal of the foreign body, the patient underwent his orthodontic treatment and was treated with a rapid maxillary expansion to improve the nasal breathing, produce space for the eruption of lateral incisors and permanent canines, and to solve the mandibular shift. The MMO after the ENT intervention was 40.93.

## 3. Discussion

Different studies have reported various symptoms of the presence of a foreign body in the external auditory canal. These objects are not meant to be there and could cause harm without medical attention [1]. This includes hearing impairment, irritation of the auditory canal, otalgia and otitis media [1]. Patients do not always disclose everything about their medical history, because they may feel ashamed or they may be afraid of being stigmatized, thus posing a challenge of attribution errors for clinicians. Often these findings could be confused with cochlear implants or radiographic artifacts due to the possible presence of earrings or piercings not paid attention to.

In this particular case, the patient’s condition remained unfound for a long time apart from a neglected hearing loss, allegedly attributed to his intellectual condition and symptom paucity.

At present, there are no studies in the literature that describe cases of incidental findings of foreign bodies through lateral cephalogram.

Lateral cephalograms are widely used for orthodontic diagnosis and the interim evaluation of skeletal and dental structures in young patients. It exposes patients to a very low radiation dose, even compared to an orthopantomography. The characteristic of interim evaluation may also make it useful, in particular in patients with intellectual disabilities and ADHD, to assess any inexplicable conditions that may involve a foreign object placed in the head and neck region. Although object location may be unprecise, it can also be more precisely located through other instrumental examinations and/or objective examination. 

In the present case report, the patient presented a hearing impairment probably due to the presence of the foreign body into the external auditory canal. It is interesting to report that the scientific literature showed that subjects presenting maxillary transverse deficiency are 3.5 times more affected by conductive hearing loss than patients with normal maxillary dimension [24]. Rapid maxillary expansion (RME) has been demonstrated to have positive and significant improvements in hearing and normal function of the eustachian tube in patients presenting maxillary hypoplasia [25]. During their clinical examination, the orthodontist should consider the relationship between conductive hearing loss (CHL) and maxillary constriction in growing children in order to consider RME treatment to restore a correct palatal anatomy [25].

Pediatric dentists and orthodontists must therefore pay particular attention to the evaluation in lateral cephalograms and panoramic radiographs of any suspicious findings and analyze all structures included in the image, since 75 percent of patients with ear foreign bodies are about eight years old or younger and may be, at least at first, asymptomatic. However, these patients could develop serious complications if not taken care of [7,10]. The real diagnostic challenge in these cases lies within the ability to diagnose foreign bodies, because some patients do not want or are not able to disclose everything about their medical history. Dental practitioners should therefore carefully examine all anatomical structures recorded in any diagnostic exam, and they should not ignore those that apparently have no relation to their purposes. Foreign bodies can lead to severe side effects if left untreated; a missed diagnosis could lead to a malpractice lawsuit against the clinician.

The radiopaque foreign object of ovoid shape in the presented case was discovered on pre-orthodontic radiographs. Although the patient denied self-insertion of a foreign object, he referred to a hearing impairment for the last 2 years that was never investigated. Radiographs made the orthodontist decide to further investigate and to refer the young patient to an ear, nose and throat specialist.

## 4. Conclusions

This case report highlights the diagnostic challenges that dentists may face, especially orthodontist and pediatric dentists, while visiting patients for the first time or during follow-up visits, especially if the patient presents difficulties in communicating his problem to his parents or caregivers for any reason.

It is necessary to always be careful in evaluating incidental findings in any anatomical structure included in a radiographic exam requested for dental diagnosis and/or interim evaluation. Even a lateral cephalogram can provide valuable information for the detection of foreign bodies. Some particulars that look meaningless or appear as radiographic artifacts may be silent incidental findings that can lead to severe complications if left untreated.

## Data Availability

The data that support the findings of this study are available from the corresponding author, upon reasonable request.
